# *Agrobacterium rhizogenes*-induced soybean hairy roots versus *Soybean mosaic virus* (ARISHR-SMV) is an efficient pathosystem for studying soybean–virus interactions

**DOI:** 10.1186/s13007-019-0442-8

**Published:** 2019-05-25

**Authors:** Hua Jiang, Kai Li, Junyi Gai

**Affiliations:** Soybean Research Institute; MARA National Center for Soybean Improvement; MARA Key Laboratory of Biology and Genetic Improvement of Soybean (General); National Key Laboratory for Crop Genetics and Germplasm Enhancement; Jiangsu Collaborative Innovation Center for Modern Crop Production, Nanjing Agricultural University, Nanjing, 210095 Jiangsu China

**Keywords:** Soybean, *Agrobacterium rhizogenes*, Hairy roots, *Soybean mosaic virus*, gene function, TBSV P19 protein, SMV CP protein

## Abstract

**Background:**

*Soybean mosaic virus* (SMV), a *Potyvirus*, is the most prevalent viral pathogen of soybean that causes severe yield and seed quality reductions in world soybean production. So far, multiple resistance loci for different SMV strains have been fine-mapped while the candidate genes’ functions need to be verified. However, identification of the resistance or susceptibility genes via stable genetic transformation is time-consuming and labor-intensive, which hinders further exploration of these genes’ functions in soybean. Thus we tried to explore a rapid and efficient method for verification of SMV-related target gene function in soybean.

**Results:**

An *Agrobacterium rhizogenes* (*A. rhizogenes*) induced soybean hairy roots versus *Soybean mosaic virus* (ARISHR-SMV) pathosystem was established. The procedure is characterized with that (1) the soybean hairy roots that can be infected by SMV are induced by *A. rhizogenes* K599 using soybean cotyledons as explants, (2) the gene to be examined for its function, which may be the endogenous SMV-resistance or -susceptible gene or exogenous SMV-related gene, is transformed into soybean calluses mediated by *A. rhizogenes*, (3) the transformed calluses on explants further inoculated with the purified tester-SMV virions using pricking method under aseptic conditions, and (4) the measurement of the SMV content in positive hairy roots for evaluating the SMV-related target gene function. The procedure takes about 30 days for one cycle. Utilizing the established procedure, the soybean hairy roots induced by *A. rhizogenes* was efficiently infected by multiple different SMV strains, the SMV infectivity in soybean hairy roots can be retained at least twice successive transfer cultures and *Tomato bushy stunt virus* (TBSV) P19 promoting and SMV CP suppressing SMV infection in soybean hairy roots was demonstrated, respectively. This procedure can also be used for identification of resistance to SMV strains for soybean germplasms.

**Conclusion:**

The ARISHR-SMV is an efficient pathosystem that allows a quick and convenient identification of SMV-related target gene function in soybean.

**Electronic supplementary material:**

The online version of this article (10.1186/s13007-019-0442-8) contains supplementary material, which is available to authorized users.

## Background

Soybean originated in China is a dominant source of plant protein and oil with multiple uses in food, feed, and industrial applications in the world. It usually subjects to various challenges from biotic and abiotic stresses during its whole growth period under natural environments. Among the biotic stresses, *Soybean mosaic virus* (SMV) disease is the major threat in all soybean-production areas in the world, especially in China, and often leads to serious yield losses (ranging from 8 to 100%) and seed quality reductions [1–3]. SMV belongs to *Potyvirus* with an approximately 9.6 kb, single-stranded, positive-sense RNA genome that contains a known open reading frame encoding at least 11 proteins and 5′ viral protein genome-linked (VPg) cap and 3′ poly(A) tail [4, 5]. A mass of SMV isolates were identified worldwide. Seven SMV strains (G1–G7) isolated from germplasm collections were reported in the United States based on the responses to a set of eight differential soybean genotypes [6]. Similarly, five SMV strains (A–E) in Japan and eleven SMV strains (G1–G7, SMV-N, G5H, G7a and G7H) in Korea have been reported [7, 8]. In China, more than 4500 country-wide SMV isolates were collected and grouped into twenty-two strains based on their responses to ten differential soybean varieties at the National Center for Soybean Improvement [9–14].

Host plant resistance is an effective, economic, and environment-friendly strategy in studying resistance to SMV. One of the prerequisites to utilize host plant resistance is to screen out SMV resistance sources to be used for gene mapping, candidate gene identification and breeding for cultivars resistant to SMV. A number of independent SMV resistance loci corresponding to specific strains have been identified in some resistance sources. For instance, in the United States three independent loci, *Rsv1*, *Rsv3*, *Rsv4* and in China sixteen independent loci, *Rsc3*, *Rsc4*, *Rsc5*, *Rsc6*, *Rsc7*, *Rsc8*, *Rsc10*, *Rsc11*, *Rsc12*, *Rsc13*, *Rsc14*, *Rsc15*, *Rsc17*, *Rsc18*, *Rsc20*, *Rsc21* have been identified from different resistance sources [2, 15, 16]. However, few of these genes has been cloned and their relationship has not been fully explored yet. In addition, the breakdown of R gene-mediated resistance occurred frequently with the rapid evolution in IPs/IPRs [17–21]. Therefore, it was urgent to establish a simple and effective system for validating the candidate genes’ functions as a quick excavation of SMV resistance in facing the co-evolution between SMV and soybean.

Recently, several new methods have been developed in verification of anti-SMV candidate genes’ functions. One approach is to transform a target gene obtained from the pathogen or host into soybean through agrobacterium-mediation or particle bombardment transformation [22–24]. However, there are still problems with the soybean genetic transformation, such as lack of easy-transformed genotypes, low transformation efficiency, imperfect regeneration system, time-consuming and labor-inefficiency, etc. which hinders the functional analysis of the candidate genes. Moreover, virus-induced gene silencing (VIGS) may substitute for stable transformation to rapidly analyze the function of the candidate genes as an effective method [25, 26]. For example, VIGS vectors based on *Bean pod mottle virus* have been used to identify components of the soybean resistance response against SMV [27, 28]. Although VIGS has been proven useful for identification of candidate genes’ functions and can overcome gene redundancy in soybean [29], it involves a viral vector which could mask or alter the defense responses of infected plants. On the other hand, the use of the hairy roots system which can be induced by transformation with a gram-negative soil bacterium *A. rhizogenes* might be also an alternative approach to study gene functions [30]. The morphological characteristics of *A. rhizogenes*-induced hairy roots were similar to that of wild-type roots, characterized with fast growth and lack of geotropism and lateral branching [31, 32]. The hairy roots were widely used as a transgenic tool to study secondary metabolism and gene function in plants. Lozovaya et al. [33] reported that down regulation of chalcone synthase or isoflavone synthase modified the phenolic metabolism in soybean hairy roots. In aspect of abiotic stress, soybean hairy roots were also applied to explore the genes responsible for salt and drought stress [34, 35]. While for biotic stresses, it has been used for propagating soybean parasites or pathogens and for testing interactions between soybean and the parasite or pathogen, for example, the soybean cyst nematode [36–38]. However, since SMV is a leaf disease, until now this approach has not been used in the study for SMV resistance yet.

The present study aimed at (1) establishing a ARISHR-SMV pathosystem that allows a rapid and effective identification of SMV-related target gene function in soybean under aseptic conditions, (2) demonstrating whether soybean hairy roots can be efficiently infected by different SMV strains, and (3) utilizing the established ARISHR-SMV pathosystem to confirm the biological function of TBSV P19 and SMV CP protein on SMV infection in soybean hairy roots.

## Materials and methods

### Plant materials and SMV strains

Soybean cultivar Williams and *NN1138*-*2* were used for *A. rhizogenes*-mediated soybean cotyledon transformation and propagating SMV, respectively. Soybean plants (*NN1138*-*2*) were grown at 25 °C with a 16 h photoperiod in a greenhouse and the unifoliolate leaves of 10-day-old plants were used for virus inoculation. There are 9 strains (SC3, SC7, SC8, SC9, SC10, SC11, SC13, SC15 and SC18) tested in the present study, which were provided by the National Center for Soybean Improvement (Nanjing Agricultural University, Nanjing, China).

### Culture of *A. rhizogenes*

The wild-type or transformed *A. rhizogenes* strain K599 was cultured in the LB medium (50 μg/ml kanamycin) and was further shaken in 4 ml LB broth (50 μg/ml kanamycin) at 200 rpm for 24 h at 28 °C. Before inoculation, the cultures were centrifuged at 4000 rpm for 5 min and then the tight pellets were gently re-suspended in 10 mM MgCl_2_. The bacterial suspension was diluted to OD600 = 0.5 with 100 mM MgCl_2_ containing 150 μM acetosyringone and gently shaken at 45 rpm for at least 30 min at 22 °C before use.

### *Agrobacterium rhizogenes*-mediated soybean cotyledon transformation

The transformation of soybean cotyledons were performed essentially as the described procedures by Subramanian et al. [39] with some modifications. The mature soybean seeds (Williams) were surface-sterilized for 6 h using chlorine gas generated by mixing 3.5 ml of 12 N HCl with 100 ml sodium hypochlorite solution. The sterilized seeds were soaked overnight in sterilized water and the seed coats were carefully removed. The unshelled seeds were further germinated on agar (0.7%) medium in a plant growth incubator with a 16 h photoperiod. When cotyledons became green and just began to separate (after approximately 4 days), the intact cotyledons were harvested and cut into a roughly small circular (about 5 mm diameter) at the petiole end to be used for *A. rhizogenes*-mediated transformation. The cotyledon pieces were transferred to White’s medium in petri plates and immediately treated with 20 μl of the freshly prepared *A. rhizogenes* suspension. The plates were then wrapped in Parafilm and placed in an incubator at 25 °C under dark culture. After 10 days, the calluses grown from the incubated cotyledon pieces were used for virus inoculation.

### SMV purification and inoculation of callus with virions

Purification of SMV and determination of the viral concentration were performed as described by Li and Pu [40]. The molecular weight of the capsid protein was estimated by SDS-PAGE on a 3.5% stacking gel and a 12% resolving gel. After electrophoresis for 2 h at 120 V at room temperature, the proteins were stained with coomassie brilliant blue. Inoculation of calluses were done aseptically by pricking the callus tissues with a sterile needle, adding 10 μl 0.05 M PB buffer or the purified SMV virions (50 μg/ml) to the wound sites. The inoculated explants were placed onto White’s medium and further cultured for growing soybean hairy roots.

### Serological determination, RT-PCR detection and back-inoculation assays of soybean

The enzyme linked immunosorbent assay (ELISA) was used for serological determination of SMV infection. The SMV antiserum (polyclonal rabbit antibodies) was purchased from ACD Inc. (cat #V094-R1, Beijing, China) and the manufacturer’s instructions were followed for the next steps. The total RNA was extracted from soybean hairy roots using RNA isolation kit (Tiangen, Beijing, China) following the manufacturer’s instructions. The quality and concentration of RNA were checked using agarose gel electrophoresis and a spectrophotometer (Nanodrop 2000, Thermo Fisher Scientific), respectively. The first-strand cDNA was synthesized using M-MLV reverse transcriptase (Invitrogen) as recommended by the manufacturer, then the 731 bp viral fragment was amplified with SMV *CP* gene specific primers (forward primer 5′-GAAGGAGACATGGATGCAG-3′ and reverse primer 5′-CTTGCAGTGTGCCTTTCAG-3′), the soybean *EF1α* gene was used as the internal control and the forward primer 5′-AAGTCTGTTGAGATGCACCA-3′ and reverse primer 5′-GCCTGTCAATCTTGGTCAAG-3′ were used to amplify the 277 bp fragment. The soybean hairy roots grown from SMV-inoculated calluses were homogenized in 0.01 M phosphate buffer (PH 7.4) in different grinders and the tissue homogenates were back-inoculated the soybean *NN1138*-*2* through mechanical inoculation.

### Construction of the TBSV *P19* and SMV *CP* overexpression vectors and transformation of *A. rhizogenes*

The *P19* sequence of *Tomato bushy stunt virus* (TBSV) [41] and the *CP* sequence of SMV SC15 strain was cloned into pEarleyGate103 [42] using gateway method, respectively. Likewise, the green fluorescent protein (GFP) derived from pEarleyGate103 was cloned into pEarleyGate202 [42] and this recombinant vector served as control. Additional file 1: Fig. S1 showed the gene arrangement in the constructs. Primers required to construct these vectors are listed in Additional file 2: Table S1. The constructed vectors validated by sequencing were transformed into *A. rhizogenes* K599 strain by electroporation.

### Identification of transgenic hairy roots

The genomic DNA was extracted from soybean hairy roots by cetyltrimethylammonium bromide method with minor modifications [43]. Insertion of the *P19* and *CP* gene into soybean genome was confirmed by PCR using the genomic DNA of hairy root transformants, respectively. Forward primer 5′-ATGGAACGAGCTATACAAGGAA-3′ and reverse primer 5′-CTCGCTTTCTTTTTCGAAGGT-3′ were used to amplify the 516 bp *P19* fragment. Forward primer 5′-GAAGGAGACATGGATGCAG-3′ and reverse primer 5′-CTTGCAGTGTGCCTTTCAG-3′ were used to amplify the 731 bp *CP* fragment. The GFP fluorescence of hairy roots transformed with the vector control, P19 or CP overexpression vector were monitored under stereo fluorescence microscope (OLYMPUS MVX10, Japan). The proteins of GFP-positive transgenic hairy roots were extracted and the protein concentrations were determined by the Bradford assay procedure using bovine serum albumin as the standard [44]: 10 μg protein samples were separated with 10% SDS-PAGE and then transferred onto a polyvinylidene difluoride membrane (Immobilon). Anti-GFP monoclonal antibody (1:3000; Sigma-Aldrich) and IRDye 800CW-conjugated goat (polyclonal) anti-mouse IgG (1:10,000; H + L; LI-COR Biosciences) secondary antibodies were used to further evaluate the gene expression in soybean hairy roots. The membranes were visualized using a LI-COR Odyssey scanner with excitation at 700 and 800 nm.

### Real-time quantification of viral RNA

The quantitative real-time polymerase chain reaction (qRT-PCR) was performed using a 7500 Real-time PCR system (Applied Biosystems) in conjunction with SYBR green reagents (TAKARA, Dalian, China). The qRT-PCR analysis of *P3* gene of SMV strain SC15 was carried out using the following primers: 5′-GAAAGTTGTAGAAGAAAGCAG-3′ (forward) and 5′-ACAAAGGCTCGCACCGATGC-3′ (reverse). The soybean *ELF1β* was used as an internal control with the following primers: 5′-GTTGAAAAGCCAGGGGACA-3′ (forward) and 5′-TCTTACCCCTTGAGCGTGG-3′ (reverse), in a 20 μl reaction, including 20 ng cDNA, 0.4 μl of each 10 μM primer, 0.4 μl ROX Reference Dye П (50×), 10 μl Premix Ex Taq (2×) and 6 μl dH_2_O. Three technical replications in each of the two biological replicates for each sample were taken for real-time PCR analysis. The relative RNA accumulation were calculated using 7500 software (version 2.0.6) supplied by the manufacturer.

### Statistical analyses

The ELISA and qRT-PCR data were analyzed for their means, standard deviations and single-factor analyses of variance using the SPSS software (version 18.0). The Fisher’ protected least significant difference tests were performed for comparisons between treated group mean and control group means using the significance level of α = 0.05.

## Results

### Establishment of the ARISHR-SMV pathosystem

**Fig. 1:**
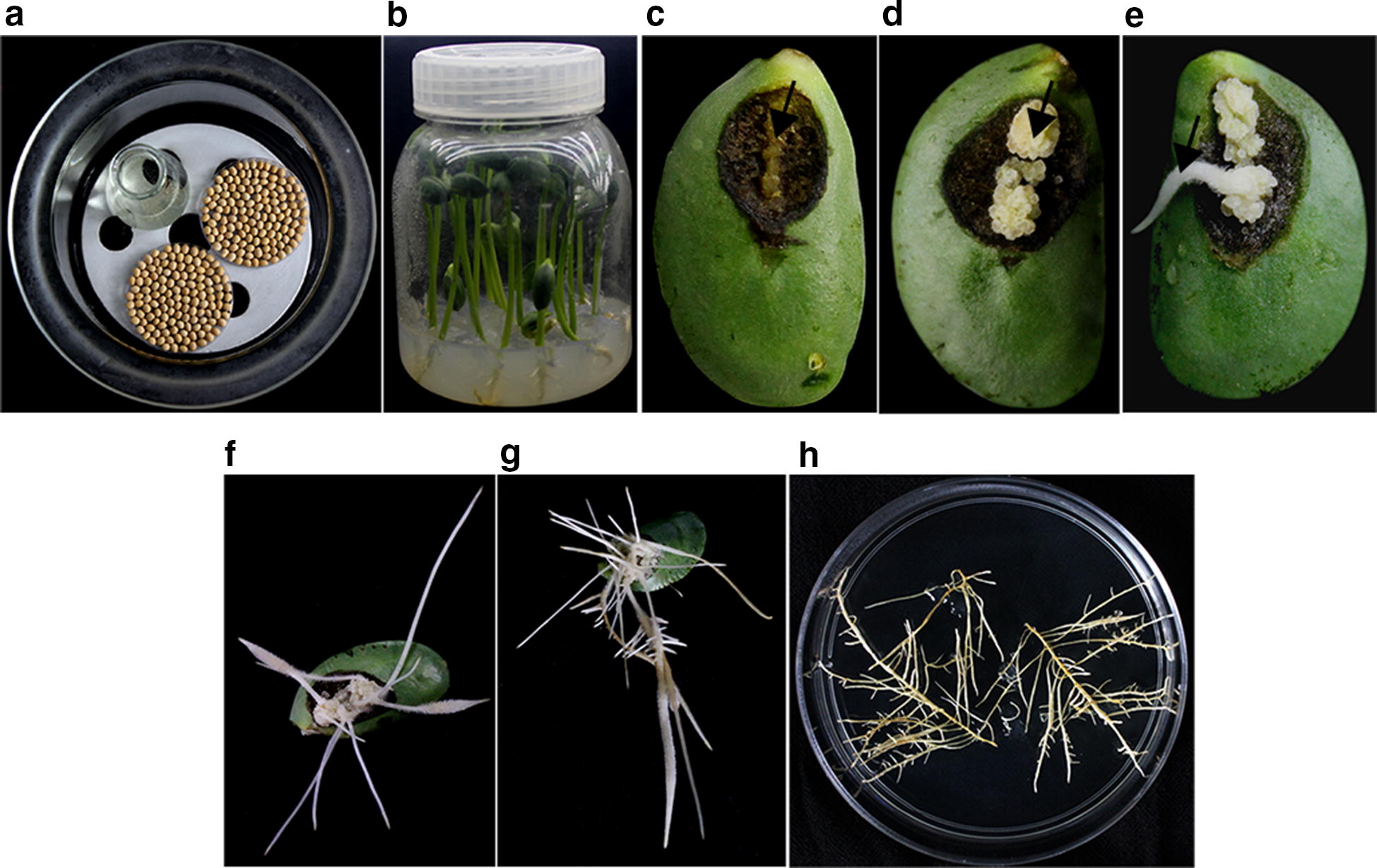
The transformation procedure and culture of soybean hairy roots under aseptic conditions. **a** The spotless soybean seeds were surface sterilized using chlorine gas in a desiccator. **b** The peeling seeds grow to about 6 cm high in 7% agar medium. The performance of cotyledon tissues on 4 days (**c**), 10 days (**d**), 12 days (**e**), 15 days (**f**), and 19 days (**g**) after infection with *A. rhizogenes* strain K599. **h** Soybean hairy roots grown from calluses were cut and transferred to White’s medium

The induction and culture of soybean hairy roots under aseptic condition is shown in Fig. 1. The processes are as the following: the soybean seeds are placed into a desiccator and surface-sterilized using chlorine gas (Fig. 1a); the peeled seeds germinate on 7% agar medium and the seedlings grow to about 6 cm in height, while the two cotyledons have not yet unfolded on about the 4th day (Fig. 1b); a ridge of callus emerges along the severed vein after inoculating the cut surface with *A. rhizogenes* strain K599 on about the 4th day (Fig. 1c); the yellowish-white callus proliferates along the vein on the 10th day (Fig. 1d) and the hairy roots begin to form on the 12th day (Fig. 1e); then abundant hairy roots form on the 15th day (Fig. 1f), with more new hairy roots begin to emerge (Fig. 1g); and the hairy roots cut from cotyledons grow strongly on White’s basal medium (Fig. 1h).

**Fig. 2:**
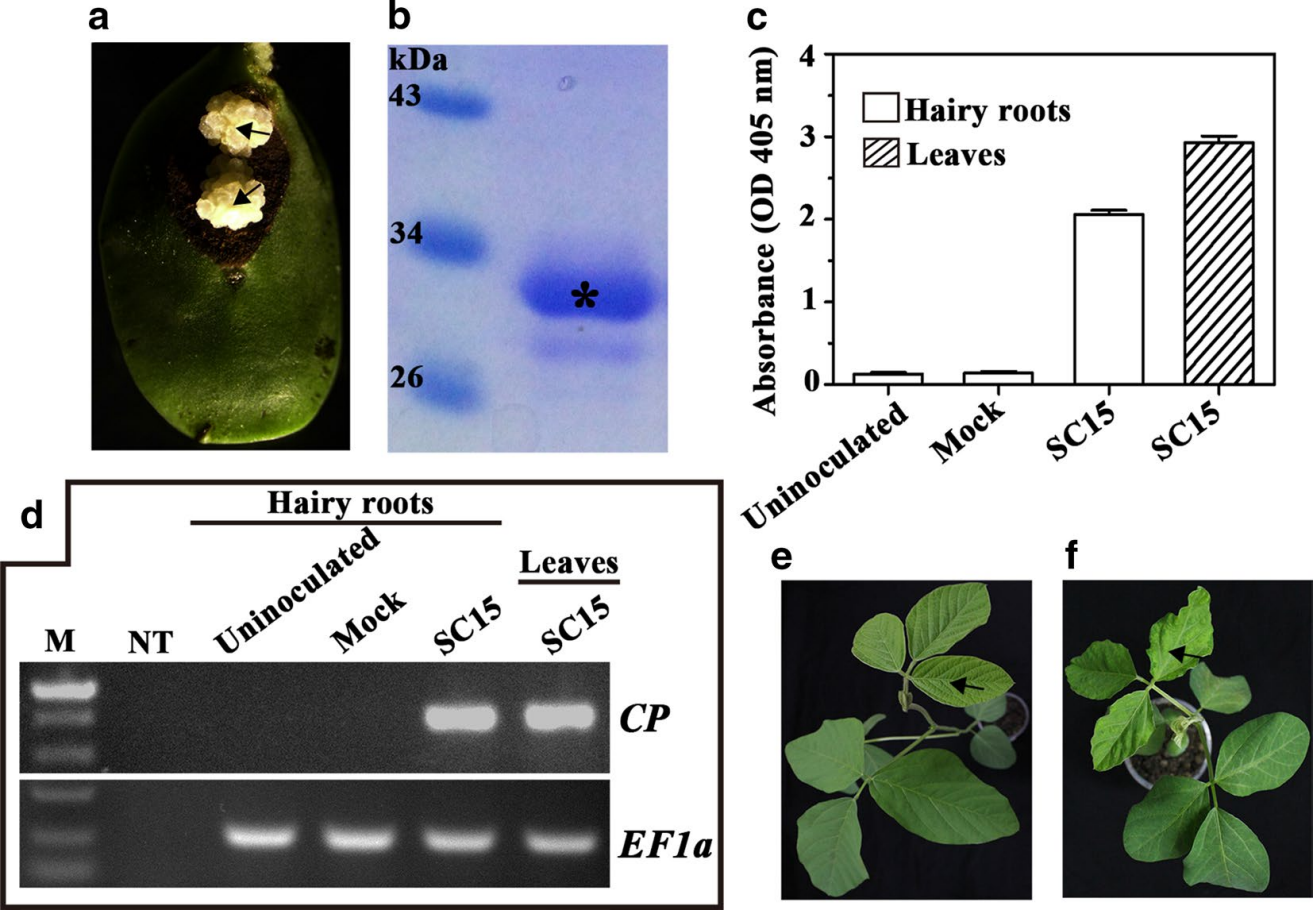
Calluses inoculation process with SMV and back-inoculation soybean assays with hairy roots sap. **a** 10 days yellowish-white calluses were pricked with a fine sterile needle at black arrow position. **b** SDS-PAGE electrophoresis of the purified SMV strain SC15 (an asterisk indicates the positions of SMV capsid protein). **c** ELISA detection of SMV coat protein in un-, Mock- and virions-inoculated soybean hairy roots with diseased soybean leaves at 14 dpi. The error bars represent standard deviations with N = 10. **d** Accumulation of viral RNA were measured by RT-PCR at 14 dpi. **e**, **f** Plants were back-inoculated with sap from hairy roots grown from Mock- and SMV-inoculated calluses, respectively, and the photographs were taken at 14 dpi

**Fig. 3:**
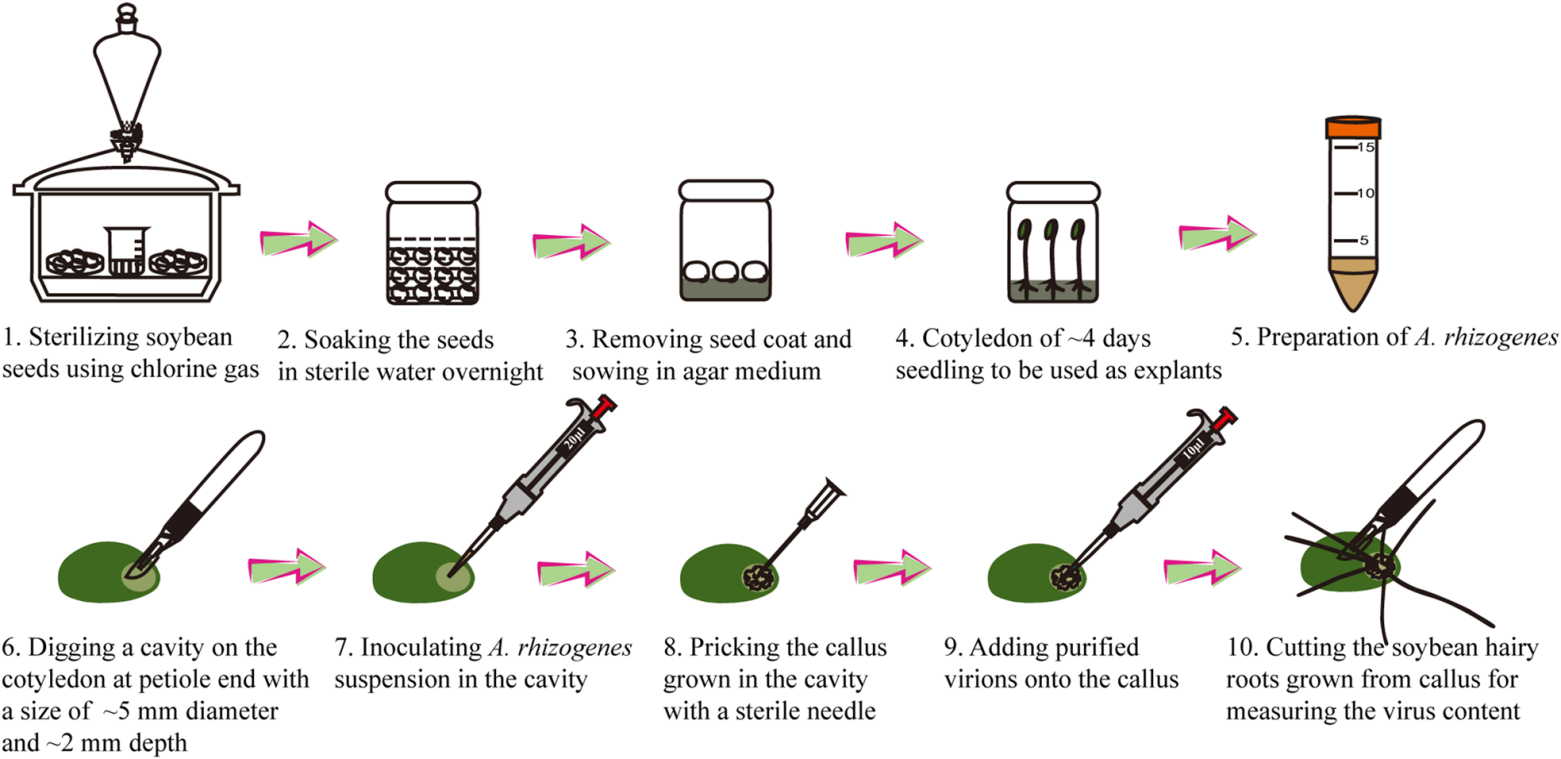
Schematic view of the established ARISHR-SMV pathosystem

To establish the ARISHR-SMV pathosystem, the soybean calluses induced by *A. rhizogenes* K599 on soybean cotyledon explants (for example, that of the cultivar Williams) are used for SMV inoculation, while the purified SMV strain (for example, SC15) virions are used as tester-inoculum. Whether the virus exist or not in hairy roots grown from SMV-inoculated calluses are measured. As shown in Fig. 2, the yellowish-white calluses were pricked with a fine sterile needle at the black arrow position in Fig. 2a and further inoculated with mock or the purified SMV virions as shown in Fig. 2b. After 14 days post inoculation (dpi), the extracts of hairy roots grown from mock- or SC15-inoculated calluses were assayed using ELISA with polyclonal antibodies to detect SMV coat protein. This revealed that SMV was present at high concentrations in the hairy roots grown from these calluses inoculated with SC15, even the viral content levels in soybean hairy roots are less than those in leaves (Fig. 2c). Meanwhile, the presence of viral RNA in hairy roots was measured by RT-PCR using SMV-specific primers. The agarose gel electrophoresis showed the presence of the 731 bp amplicon in hairy roots inoculated with strain SC15 (Fig. 2d). Moreover, the susceptible soybean line *NN1138*-*2* back-inoculated with the sap from SMV infected hairy roots developed typical mosaic symptoms (Fig. 2f) and no symptoms were observed in mock control following mechanical inoculation at 14 dpi (Fig. 2e). As shown in Fig. 3, the entire process takes about 1 month, including 5 days for preparation of the soybean explant, 10 days for growth of soybean callus with which the growth status can just fit for SMV inoculation and 15 days for the period from virus inoculation to harvest of the infected soybean hairy roots.

### Multiple SMV strains can infect soybean hairy roots using the ARISHR-SMV pathosystem

To know whether other SMV strains can infect hairy roots through this ARISHR-SMV pathosystem method, the purified SMV strains SC3, SC7, SC8, SC9, SC10, SC11, SC13 and SC18 virions were used as tester to inoculate soybean calluses, respectively, and the hairy roots grown from these calluses were further assayed with ELISA. The results revealed that SMV was detected in all the soybean hairy roots grown from the calluses inoculated with different SMV strains (Table 1). All SMV-infected soybean hairy roots gave 85.7–100% infection in infectivity assay through back-inoculating the soybean line *NN1138*-*2* (Table 1). As expected, the mock-inoculated hairy roots exhibited negative reactions in serological tests (Table 1). Taken together, the above results suggested that all the 9 SMV strains can be applied to the ARISHR-SMV pathosystem. Besides, the pathosystem can be used also for identification of resistance to these 9 SMV strains in soybean germplasms.

**Table 1:** Serological tests of *A. rhizogenes*-induced soybean hairy roots and the performance of back-inoculating soybean with sap from hairy roots, as determined by DAS-ELISA and infectivity assays, respectively

SMV strains	ELISA (OD 405 nm)^a^	FC^b^	Infected/inoculated plants	
			II^c^	S (%)^d^
SC3	2.11*	19.18 (+)	18/19	94.7
SC7	2.27*	20.64 (+)	18/18	100
SC8	1.86*	16.91 (+)	14/16	87.5
SC9	2.31*	21.00 (+)	15/15	100
SC10	1.91*	17.36 (+)	21/21	100
SC11	1.75*	15.91 (+)	18/20	90.0
SC13	2.05*	18.64 (+)	17/17	100
SC18	1.92*	17.45 (+)	18/21	85.7
Mock	0.11	1.00 (−)	0/23	0

Each data point represents total number of hairy roots or plants from two replications

*Represents significance difference between treated group and control group of the individual means at 0.05 probability level

^a^Mean absorbance of extract of soybean hairy root at 405 nm

^b^The mean OD 405 nm value of each sample and the mean OD 405 nm value of negative control ratio. (+): positive for SMV; (−): negative for SMV

^c^Infectivity was determined based on the number of infected plants/total number of plants inoculated

^d^Average % infection for each SMV strain

### Retention of SMV infectivity in hairy roots with successive transfer cultures

To further develop this ARISHR-SMV pathosystem method, the key aspects of the interaction between SMV and soybean hairy root cells in vitro was investigated. The maintenance of viral stability and infectivity in hairy roots was especially concerned. The SMV infectivity was measured through back-inoculating soybeans with the sap obtained from soybean hairy roots which were transferred to White’s medium one or two times. The results showed that the virus in a majority of hairy roots was infective after one or two successive transfer cultures, it produced 70.2–86.6% infected plants on average for different SMV strains (Table 2). In addition, viral infectivity rate was similar as the results between the two transfers (Table 2). As expected, no virus was detected in the non-inoculated control by infectivity assay (Table 2). The above results indicated that the SMV infectivity in soybean hairy roots can be retained at least twice successive transfer cultures.

**Table 2:** Persistence of soybean mosaic virus in *A. rhizogenes*-induced soybean hairy roots after one (2 weeks in culture) and two (4 weeks in culture) transfers, as measured by infectivity tests

SMV strains	First transfer		Second transfer		Average S (%)^c^
	II^a^	S (%)^b^	II^a^	S (%)^b^	
SC3	16/21	76.2	15/19	78.9	77.6
SC7	21/25	84.0	19/23	82.6	83.3
SC8	15/17	88.2	17/20	85.0	86.6
SC9	19/25	76.0	21/26	80.8	78.4
SC10	20/25	80.0	18/21	85.7	82.9
SC11	14/19	73.7	14/21	66.7	70.2
SC13	22/26	84.6	17/20	85.0	84.8
SC15	21/25	84.0	20/23	87.0	85.5
SC18	15/21	71.4	16/21	76.2	73.8
Mock	0/26	0	0/26	0	0

Each data point represents total number of plants from two replications

^a^Infectivity was determined based on the number of infected plants/total number of plants inoculated

^b^Average  % infection of every transfer for each SMV strain

^c^Average  % infection of two transfers for each SMV strain

### Validation of TBSV-P19 promoting and SMV-CP suppressing SMV infection in soybean hairy roots using ARISHR-SMV pathosystem

**Fig. 4:**
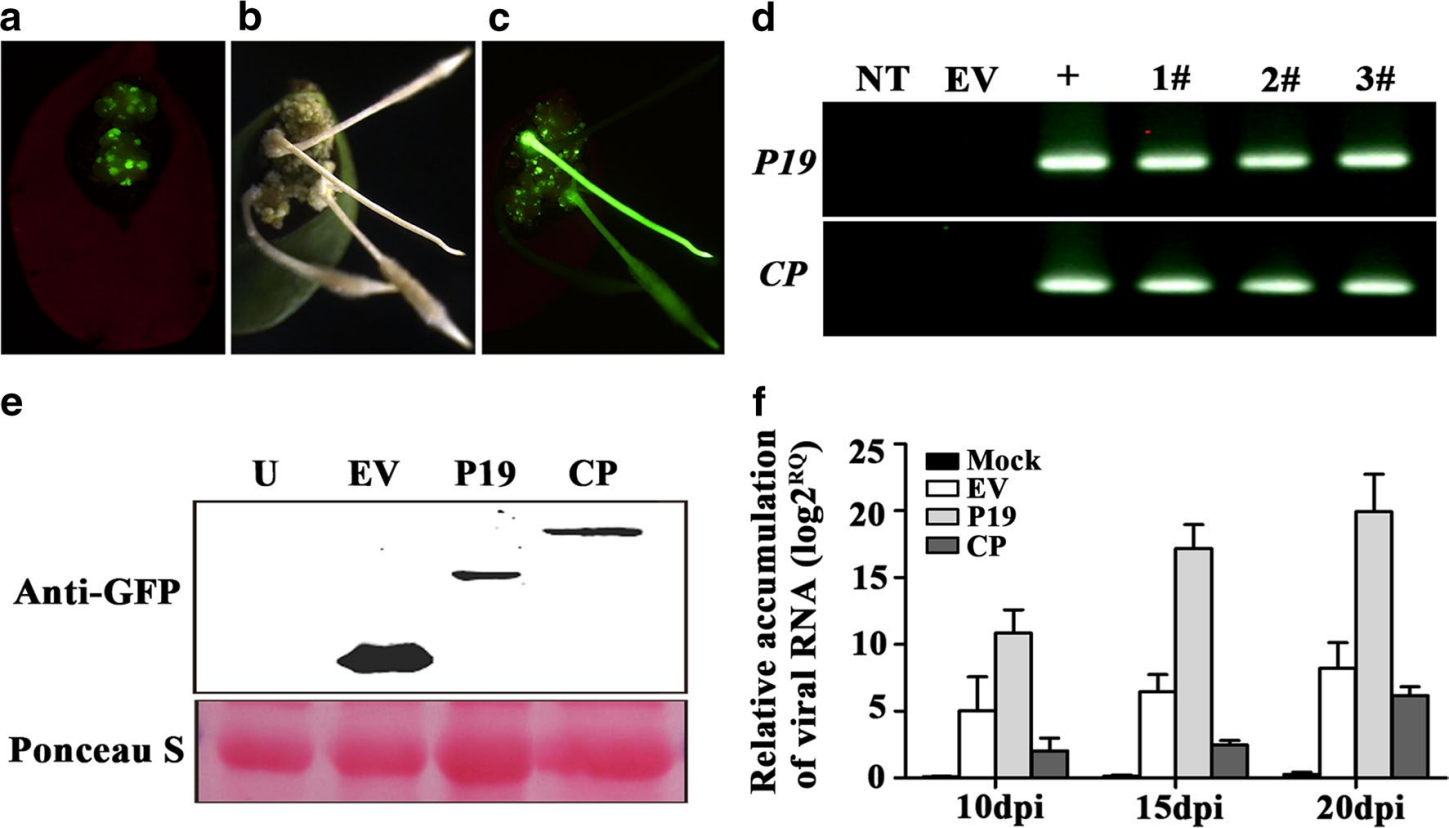
Verification of *P19* and *CP* transformation events and the effect of P19 and CP on SMV accumulation in soybean hairy roots, respectively. **a** Soybean calluses were induced by K599 carrying control or destination vector on 10 days under stereo fluorescence microscope. Soybean hairy roots of a cotyledon transformed with control or destination vector are shown on 15 days under white light (**b**) and long wavelength UV light with a GFP barrier filter (**c**). **d** gDNA PCR detection of *P19* and *CP* amplicon in transgenic hairy roots. NT: no template; EV: vector control; +: plasmid; 1#, 2#, 3#: different lines of transgenic hairy roots. **e** Western blot confirm the presence of P19 and CP protein in hairy roots. U: no plasmid; EV: vector control; P19: P19 plasmid; CP: CP plasmid. **f** Amount of viral RNA accumulating in hairy roots at 10, 15, and 20 dpi. The error bars indicate standard deviations calculated from three technical replications

The P19 of TBSV was a suppressor of gene silencing in plants [41, 45], which prevents the onset of post-transcriptional gene silencing that is natural plant antiviral mechanism in eukaryotes, and promotes pathogen infection in the tissue-specific expression of *P19* gene. On the other hand, the previous research reported that overexpression of SMV *CP* gene in soybean enhanced the transgenic plants’ resistance to SMV [23, 46]. The mechanism of pathogen-derived resistance (PDR) was reported involving post-transcriptional RNA silencing [47, 48] or the CP itself [49, 50]. In order to test the ARISHR-SMV pathosystem, we introduced the TBSV *P19* and SMV *CP* gene into the soybean hairy roots separately and purified the SMV strain SC15 as tester. After just 10 days, transformation of the callus with appropriate vector control, P19 and CP overexpression vector were monitored with fluorescence of the GFP marker (Fig. 4a). The callus which can produce more than five fluorescent spots was used to inoculate strain SC15 (Fig. 4a), otherwise, it was discarded. Among the 4 roots shown in Fig. 4b, two of them had been transformed by K599 carrying a P19 or CP overexpression vector (Fig. 4c). The remained two might come from emerged adventitious roots or the transformation of the wild-type K599 strain. We observed that approximately 30% of the hairy roots were successfully transformed with P19 or CP overexpression vector on average. The time or efficiency of root formation was not changed when vector control, P19 or CP overexpression vector presents in K599. Meanwhile, the gDNA PCR results showed the presence of the 516 bp and 731 bp amplicon in transgenic hairy roots using specific primers of *P19* or *CP* gene, respectively (Fig. 4d). The western blot results (Fig. 4e) confirmed the fluorescence observed in Fig. 4c. For determining whether P19 promoted and CP suppressed the accumulation of SMV strain SC15 in transgenic hairy roots, the virus content in positive hairy roots was measured at 10, 15 and 20 dpi using qRT-PCR, respectively. The results revealed that SMV concentration was significantly higher in *P19* transgenic hairy roots and lower in *CP* transgenic hairy roots than that in control at 10, 15 and 20 dpi, respectively (Fig. 4f). Taken together, the ARISHR-SMV pathosystem has certain potential for rapid and effective identification of exogenous SMV-related candidate gene function, or endogenous soybean candidate resistance gene or susceptibility gene function in soybean under aseptic conditions. Meanwhile, pre-overexpression of the *P19* in soybean hairy roots was an optional way of enhancing the ability of SMV infection.

## Discussion

### The ARISHR-SMV pathosystem is a rapid and efficient method for verification of SMV-related target gene function in soybeans

In recent years, the hairy roots system has been extensively used to study the gene functions of soybeans in different cases. For example, Li et al. [34] reported that *GmNAC15* overexpression in hairy roots enhanced salt tolerance in soybean. Wang et al. [35] reported that *GmWRKY27* interacted with *GmMYB174* to reduced the expression of *GmNAC29* for drought tolerance in soybean hairy roots. Liu et al. [36] identified a soybean cyst nematode resistance gene using hairy roots system which can be used to reduce soybean yield losses. However, soybean hairy root system has not been used in the study for SMV infection. In the present study, the ARISHR-SMV pathosystem was established and can be used for studying soybean-virus interactions in hairy roots, which can broaden the research scope of hairy roots system (Figs. 1, 2, 3, 4). Recently, Morriss et al. [37] developed an interaction system between soybean hairy roots and aphid that allowed effective aphid feeding and validation of aphids resistance genes. Combining the present results with the reports of Morriss et al. [37], all three factors (soybean, SMV and aphid) would be integrated into the hairy roots system to study the interaction relationship among them. Meanwhile, the ARISHR-SMV pathosystem just need to take about 1 month for the entire flow and allowed rapid identification of the target genes responsible for SMV resistance or susceptibility, which is more efficient than the normal soybean genetic transformation when screening a mass of SMV-related target genes’ functions. Prior to this, Jiang et al. [51] reported a SMV variant belonging to SMV SC7 strain caused different diseases in *Glycine max* and *Nicotiana benthamiana*, and the interaction system between SMV and *Nicotiana benthamiana* was an ideal model for investigating the resistance genes to SC7 strain, but it is limited merely to the specific strain. Compared with the results of Jiang et al. [51], the newly established ARISHR-SMV pathosystem can be applied for at least 9 SMV strains, therefore, it is characterized with multiple uses in the study of SMV resistance or susceptibility genes (Tables 1 and 2). Latterly, two methods of transiently expressing a target gene using *Agrobacterium* infiltration in soybean leaf tissue were developed, however, the small amount of infectious tissues and the low levels of replicated experiments may have limited their practical implications [52, 53]. Additionally, Wu and Hanzawa [54] reported that a protocol for the isolation of soybean protoplasts and the application to transient expression studies, while it is not suitable for observation of long term cellular processes. In ARISHR-SMV pathosystem, induction and culture of soybean hairy roots are under aseptic condition, the transformation efficiency of hairy roots is controllable and SMV infectivity can be maintained in hairy roots with successive transfers (Figs. 1, 2, 3, 4, Tables 1, 2). Thus, the ARISHR-SMV pathosystem can be used for observation of gene effect during long-term process of SMV infection which provide convenience for the study on disease-related candidate gene functions. Besides, SMV and its counterpart soybean hairy roots fit well the Invasion Model in which host receptors, termed invasion pattern receptors (IPRs) detect either an externally encoded or modified-self ligand that indicates invasion, termed invasion pattern(s) [IP(s)] [21] and therefore the ARISHR-SMV pathosystem can be used to excavate not only new IPRs but also new IPs in SMV-soybean competition, which supply an opportunity for rapid study the molecular mechanisms of soybean resistance to SMV.

### The ARISHR-SMV pathosystem can be used also for SMV resistance identification of soybean germplasm

Different from the previous method of resistance identification of soybean cultivars to SMV, which evaluating the whole performance of the inoculated plants to assess the resistance or susceptibility of soybean germplasm to SMV [55]. The present study utilizing *A. rhizogenes* to induce calluses of a certain soybean genotype for inoculating the purified SMV virions and estimating varietal resistance according to the SMV presence of the inoculated soybean calluses or hairy roots (Figs. 1, 2, 3). With this method, a soybean plant (genotype) can be used for multiple SMV strains since a callus can be separated into multiple parts while the conventional method has to spend one plant for one strain, which is impossible if each plant is a unique genotype. Moreover, the previous method was greatly affected by climate, especially the outside temperature, therefore, it cannot be carried out in the whole year, while the ARISHR-SMV method can save manpower and space costs, and is free from the influences of temperature and humidity, therefore, can be performed at any time with more flexibility. However, this method also has some shortcomings in SMV resistance identification of soybean germplasm, i.e., the infection of *A. rhizogenes* will induce plant defense response and changes in hormone levels, which could lead to a synergistical effect with SMV infection [56]. As a consequence, the results of resistance identification of soybean cultivars to SMV will be possible to appear false positive. Even so, the ARISHR-SMV pathosystem still supply an additional and aided approach for SMV resistance identification in soybean. For a broad application of this new method, whether soybean hairy roots can be infected by more SMV strains needs to be further investigated.

## Conclusions

A pathosystem, designated ARISHR-SMV, is established for a rapid and effective identification of SMV-related target gene function, which composes of utilizing *A. rhizogenes* transformed with the target gene to induce calluses, inoculating the calluses with SMV virions and measuring the viral content in hairy roots derived from the calluses. The soybean hairy roots can be efficiently infected by different SMV strains and SMV infectivity in soybean hairy roots can be retained at least twice successive transfer cultures. In addition, this ARISHR-SMV pathosystem was successfully applied to confirm the function of P19 and CP protein on SMV infection in soybean hairy roots, respectively. This method provided a new option for investigating SMV resistance or susceptibility gene function in soybean.

## Additional files

**Additional file 1: Fig. S1.** The gene arrangement in T-DNA constructs used in this study.

**Additional file 2: Table S1** The primers are used to construct control and destination vectors.

## Data Availability

The data sets supporting the results of this article are included within the article.

## Abbreviations

SMV: *Soybean mosaic virus*
*A. rhizogenes*: *Agrobacterium rhizogenes*
ARISHR-SMV: *Agrobacterium rhizogenes* induced soybean hairy roots-*Soybean mosaic virus*
VIGS: virus-induced gene silencing
TBSV: *Tomato bushy stunt virus*
ELISA: enzyme linked immunosorbent assay
qRT-PCR: quantitative real-time polymerase chain reaction
dpi: days post-inoculation
