# Supplementary figures and images for: Agrobacterium rhizogenes-induced soybean hairy roots versus Soybean mosaic virus (ARISHR-SMV) is an efficient pathosystem for studying soybean–virus interactions
Source: Plant Methods. 2019 May 25;15:56. doi: 10.1186/s13007-019-0442-8 (PMC6534890; doi:10.1186/s13007-019-0442-8)

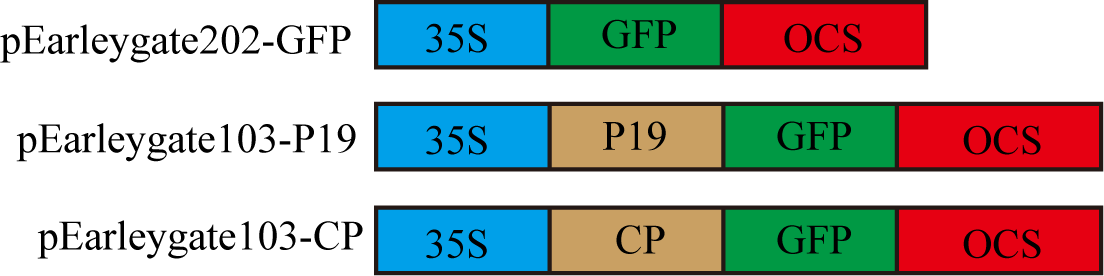

Supplement: Supplementary file 1 — Additional file 1: Fig. S1. The gene arrangement in T-DNA constructs used in this study. [file 13007_2019_442_MOESM1_ESM.tif]
