# Supplementary material for: Agrobacterium rhizogenes-induced soybean hairy roots versus Soybean mosaic virus (ARISHR-SMV) is an efficient pathosystem for studying soybean–virus interactions
Source: Plant Methods. 2019 May 25;15:56. doi: 10.1186/s13007-019-0442-8 (PMC6534890; doi:10.1186/s13007-019-0442-8)
Supplement: Supplementary file 2 — Additional file 2: Table S1 The primers are used to construct control and destination vectors. [file 13007_2019_442_MOESM2_ESM.docx]

**Table S1.** The primers are used to construct control and destination vectors.

| **Primer name** | **Primer sequence (5'to 3')** | **Purpose** |
| --- | --- | --- |
| attB1-GFP-forward primer | GGGGACAAGTTTGTACAAAAAAGCAGGCTTCACCATGGTAGATCTGACTAGTAAAGG | GFP BP recombination reaction |
| attB2-GFP-reverse primer | GGGGACCACTTTGTACAAGAAAGCTGGGTTTCAGCTAGCTTTGTATAGTTCATCC |  |
| attB1-*P19*-forward primer | GGGGACAAGTTTGTACAAAAAAGCAGGCTTCACCATGGAACGAGCTATACAAGGAA | *P19* BP recombination reaction |
| attB2-*P19*-reverse primer | GGGGACCACTTTGTACAAGAAAGCTGGGTTCTCGCTTTCTTTTTCGAAGGTC |  |
| attB1-*CP*-forward primer | GGGGACAAGTTTGTACAAAAAAGCAGGCTTCACCATGTCAGGCAAGGAGAAAGAAGGA | *CP* BP recombination reaction |
| attB2-*CP*-reverse primer | GGGGACCACTTTGTACAAGAAAGCTGGGTTCTGCTGTGGACCTATGCCCA |  |
